# Metabolic dysfunction-associated fatty liver disease and risk of nephrolithiasis: a sizeable cross-sectional study

**DOI:** 10.3389/fendo.2024.1406065

**Published:** 2025-01-21

**Authors:** Shengqi Zheng, Tianchi Hua, Guicao Yin, Wei Zhang, Xiaoxiang Wang, Lezhong Qi, Xiayong Jing, Qibing Fan, Xiaoping Yu, Yifan Li

**Affiliations:** ^1^ Department of Urology, Affiliated Hospital of Yangzhou University, Yangzhou University, Yangzhou, Jiangsu, China; ^2^ Department of Health Promotion Center, Affiliated Hospital of Yangzhou University, Yangzhou University, Yangzhou, Jiangsu, China

**Keywords:** metabolic dysfunction-associated fatty liver disease, nephrolithiasis, liver steatosis, metabolic syndrome, cross-sectional study

## Abstract

**Objective:**

Metabolic dysfunction-associated fatty liver disease (MAFLD) and nephrolithiasis are two common metabolic diseases, but their relationship has not yet been thoroughly studied. Therefore, this study aimed to explore the association between MAFLD and nephrolithiasis and to assess the effect of MAFLD on the risk of nephrolithiasis.

**Materials and methods:**

This cross-sectional study included 96,767 adults from China. All participants underwent medical examinations, including physical examinations, medical history tests, and laboratory tests. Based on ultrasound examination, participants were divided into MAFLD and non-MAFLD groups, and the severity of liver steatosis was determined based on ultrasound images. The relationship between MAFLD and nephrolithiasis was analyzed using a multivariate logistic regression model and subgroup analysis was performed.

**Results:**

The proportion of participants with MAFLD was significantly higher in the nephrolithiasis group compared to the non-nephrolithiasis group (47.70% vs. 30.45%, *P* < 0.001). Multivariate logistic regression analysis showed a significant positive association between MAFLD and nephrolithiasis (adjusted *OR*=1.38, 95% *CI*: 1.29 to 1.47). Subgroup analyses indicated that, even after accounting for various factors such as age, diabetes, hypertension, obesity, lipid profiles, and renal function, the positive association between MAFLD and an increased risk of nephrolithiasis remained consistent. Further subgroup analysis revealed that in male patients with MAFLD, the risk of nephrolithiasis increased progressively with increasing severity of liver steatosis. The adjusted multivariable odds ratios were 1.43 (95% *CI*: 1.33 to 1.53) for mild, 1.48 (95% *CI*: 1.32 to 1.67) for moderate, and 1.94 (95% *CI*: 1.47 to 2.58) for severe hepatic steatosis.

**Conclusions:**

This study found a significant positive association between MAFLD and nephrolithiasis. The risk of nephrolithiasis in males with MAFLD increased substantially with increasing severity of liver steatosis. Therefore, it is essential to strengthen prevention and screening for nephrolithiasis in individuals with MAFLD. More research is needed to elucidate the physiological and pathological mechanisms between MAFLD and nephrolithiasis.

## 1 Introduction

Nephrolithiasis is a common disease of the urinary system. It is characterized by the formation of mineral concretions, which can obstruct the urinary tract and cause significant pain and discomfort. The prevalence of nephrolithiasis ranges from 1% to 13% in different regions (1). Furthermore, the incidence is increasing annually, raising significant concerns in urology (2). The recurrence rate of nephrolithiasis is also relatively high, about 15% within one year, 35% within five years, and 50% within ten years (3). Its direct and indirect costs impose a heavy economic burden on patients and society. Therefore, it is crucial to take proactive measures to prevent the formation of nephrolithiasis and recurrence after treatment.

Nonalcoholic fatty liver disease (NAFLD) is a frequent cause of chronic liver disease, which is estimated to affect one-quarter of the adult population worldwide and is expected to increase further (4, 5). A previous meta-analysis, including seven studies and 226,541 individuals, revealed that the risk of urolithiasis was higher (*OR*=1.73, 95% *CI*: 1.24 to 2.40) in the NAFLD population than in healthy controls (6). Metabolic dysfunction-associated fatty liver disease (MAFLD) has been recommended by international consensus to replace NAFLD due to its emphasis on metabolic dysfunction in pathogenesis (7). The advantages of MAFLD over traditional NAFLD terminology have been demonstrated in several key areas, including the risk of hepatic and extrahepatic mortality, the association with disease, and the identification of individuals at risk (8, 9). Several national and international societies have adopted MAFLD due to its concise diagnostic criteria and elimination of the requirement to exclude other liver diseases (10, 11).

Although prior research has associated NAFLD with an increased risk of nephrolithiasis, the interaction between MAFLD and nephrolithiasis remains poorly defined. This study aims to elucidate the association between MAFLD and nephrolithiasis. By investigating the links between hepatic steatosis and the incidence of urinary stones, this study will provide new information to inform future research on the prevention and treatment of nephrolithiasis.

## 2 Materials and methods

### 2.1 Study population

**Figure 1:**
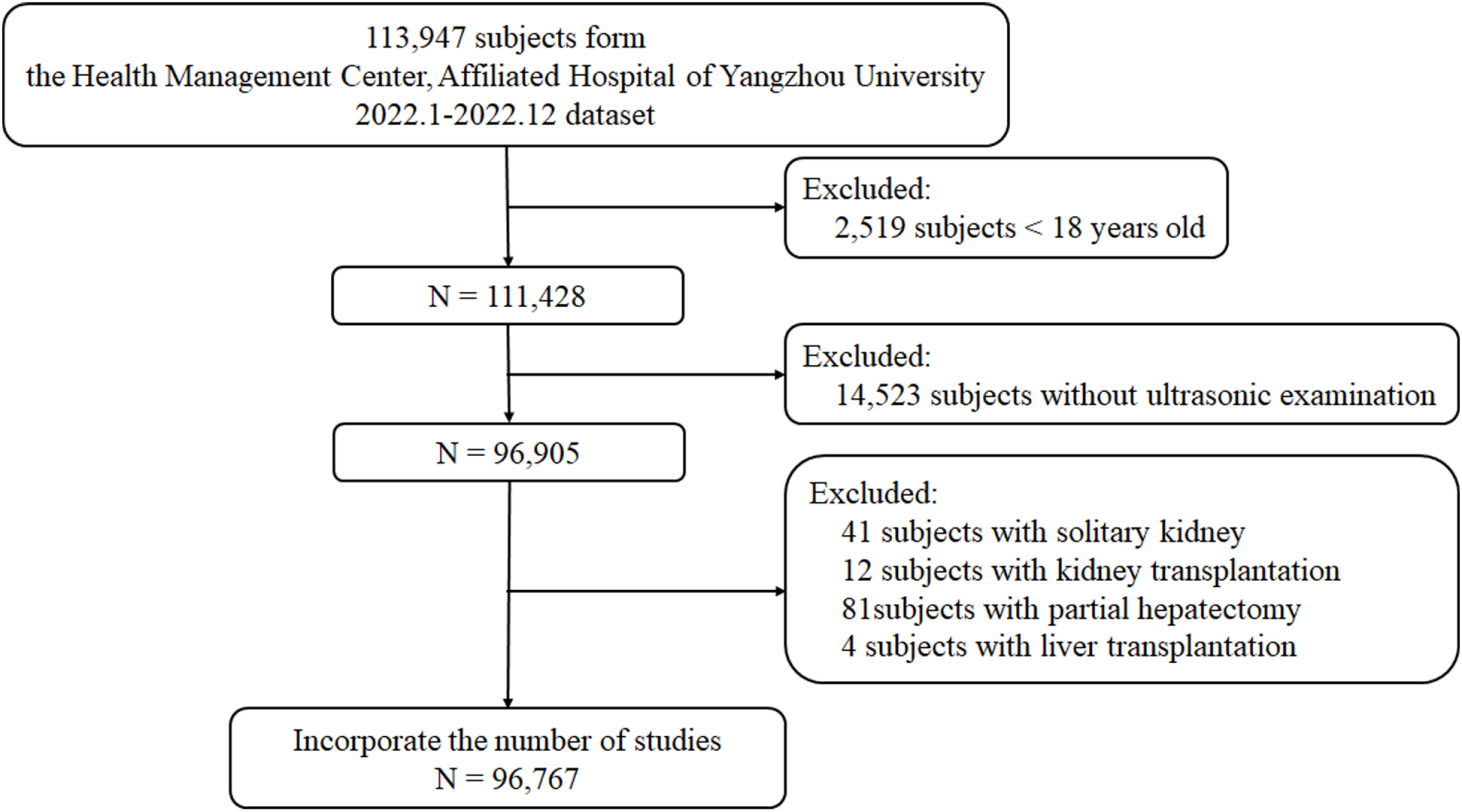
Participant enrollment and follow-up flowchart.

This cross-sectional study was conducted at the Affiliated Hospital of Yangzhou University Health Promotion Center from January 2022 to December 2022. As shown in **Figure 1**, subjects who completed the health screening (n=96,767) were recruited after excluding those under 18 years of age (n = 2,519), without ultrasound of the liver and kidney (n=14,523), or with a solitary kidney (n=41), kidney transplantation (n=12), partial hepatectomy (n=81) and liver transplantation (n=4). Due to the retrospective nature of this study, the need for informed consent was waived. This study adhered to the ethical guidelines of the Declaration of Helsinki and was approved by the Ethics Committee of the Affiliated Hospital of Yangzhou University (approval number: 2023-YKL01-13).

### 2.2 Diagnostic criteria

Ultrasonography for the diagnosis of the disease in this study was performed by experienced sonologists certified above the intermediate rank. Upon review of the ultrasound findings, nephrolithiasis and liver steatosis were diagnosed in the patient (using an abdominal convex array probe, frequency: 3.5–5 MHz, LOGIQ E9, GE, USA). Hepatic steatosis was interpreted by the presence of one or more of the following: 1) diffuse enhancement of liver echogenicity in the near field with greater echoes than the kidneys, 2) poorly visualized intrahepatic ductal structures, 3) progressive attenuation of far field echogenicity in the liver. The sonographer further classified the severity of hepatic steatosis as mild, moderate, or severe according to the criteria mentioned above, providing a comprehensive diagnosis of the patient’s condition.

MAFLD was diagnosed based on ultrasound evidence of liver steatosis and any of the following three conditions: 1) overweight/obesity, 2) type 2 diabetes mellitus and 3) metabolic dysfunction. Metabolic dysfunction was defined as the presence of at least two metabolic risk abnormalities, including 1) waist circumference ≥90/80 cm in men and women; 2) blood pressure ≥130/85 mmHg or diagnosed and treated hypertensive disease: 3) plasma triglycerides ≥1.70 mmol/L; 4) plasma HDL cholesterol <1.0 mmol/L in male or plasma HDL cholesterol <1.3 mmol/L in female; 5) prediabetes (i.e., fasting blood glucose level 5.6-6.9 mmol/L) (7).

Nephrolithiasis is diagnosed by ultrasound, a common method to detect nephrolithiasis. The presence of solid echogenic dots and clusters in the kidney that show posterior acoustic shadowing on the sonogram is a diagnostic of nephrolithiasis (12).

### 2.3 Clinical and laboratory parameters

The health promotion center professionals collected data and examined the examinees. Data included general demographic characteristics (age, sex, etc.) and history of underlying diseases (hypertensive disease, diabetes, dyslipidemia, tumor, surgical history, etc.). The hypertensive disease was defined as the presence of one of the following three criteria: systolic blood pressure ≥ 140 mmHg and/or diastolic blood pressure ≥ 90 mmHg or the participant self-reported a history of prior diagnosed hypertension or was taking anti-hypertensive medication. Diabetes was defined by the presence of one of the following: (1) self-reported history of previously diagnosed diabetes; (2) fasting glucose ≥7.0 mmol/L; and (3) self-reported use of anti-diabetic medications, including insulin.

Physical examinations included height, weight, waist circumference (WC), systolic blood pressure (SBP), diastolic blood pressure (DBP), etc. Body mass index (BMI) was calculated, $\text{BMI}=\frac{\text{weight}\left(\text{kg}\right)}{\text{height}{\left(\text{m}\right)}^{2}}$ BMI ≥ 28 kg/m^2^ is diagnosed as obesity according to the criteria for Chinese adults (13).

All subjects fasted for 12 hours overnight before their blood was drawn. All samples were routinely collected and sent to the laboratory for uniform testing using the dry chemical method (C16000, Abbott, USA). Laboratory parameters included aminotransferase (ALT), aspartate aminotransferase (AST), γ-glutamyl transpeptidase (GGT), platelets (Plt), globulin (Glo), fasting glucose (Glu), total protein (TP), total cholesterol (TC), triglycerides (TG), high-density lipoprotein cholesterol (HDL-C), low-density lipoprotein cholesterol (LDL-C), serum creatinine (SCr) and uric acid (UA) were measured from blood samples. In our study, we applied the CKD-EPI equation to estimate the eGFR (estimated glomerular filtration rate) in the Chinese population. The equation used for the eGFR calculation is as follows:


$$\begin{array}{c} \text{eGFR}=141\times \min{\left(\frac{\text{Scr}}{\kappa },1\right)}^{\alpha }\times \max{\left(\frac{\text{Scr}}{\kappa },1\right)}^{-1.209}\times 0.993^{\text{Age}}\\ \times 1.018\text{ [if female]} \end{array}$$

Scr represents the serum creatinine concentration, κ is 0.7 for females and 0.9 for males, α is -0.329 for females and -0.411 for males (14).

### 2.4 Statistical analysis

Multiple imputations were applied to evaluate the missing values. Data are presented as mean ± standard deviation or value (percentage). Qualitative data was compared using the chi-square test. Logistic regression models were used to assess the correlation between the selected variables and the occurrence of nephrolithiasis, expressed as the ratio (*OR*) (95% confidence interval [*CI*]). Covariates were included as potential confounders in the final models if they changed MAFLD estimates on nephrolithiasis by more than 10% or were significantly associated with nephrolithiasis. Models were based on age and comorbidities, including obesity, hypertensive, diabetes (model 1) and laboratory variables, including ALT, AST, GGT, TG, HDL-C, LDL-C, UA, and eGFR (model 2).

Subgroup analyzes were performed for males and females, respectively. Variables were grouped as follows: age (<60,≥60 years), hypertension (no, yes), diabetes (no, yes), obesity (no, yes), HDL-C (for females,<1.3, ≥1.3 mmol/L; for males,<1.0, ≥1.0 mmol/L), LDL-C (<3.4, ≥3.4 mmol/L), TG (<1.7, ≥1.7 mmol/L) according to the Third Report of the National Cholesterol Education Program (15), and eGFR (<90, 90-119, ≥120 ml/min/1.73 m^2^). The interactions between subgroups tests were performed using the Wald test.

A sensitivity analysis was performed between populations with missing data and multiple imputation (MI). All data were analyzed with the use of the statistical packages R (The R Foundation; http://www.r-project.org; version 4.2.0) and EmpowerStats (www.empowerstats.net, X&Y solutions, Inc. Boston, Massachusetts). *P*<0.05 was set as statistically significant.

## 3 Results

### 3.1 Baseline characteristics of the patients

Among the 96,767 participants, 56,174 were male (58.05%), and 40,593 were female (41.95%). **Table 1** indicates the baseline characteristics of the study participants. Of whom, 30,602 individuals had MAFLD, and 6,579 individuals had nephrolithiasis, leading to an overall prevalence of 31.62% for MAFLD and 6.80% for nephrolithiasis. Compared to individuals without nephrolithiasis, those with nephrolithiasis were older (49.30 ± 13.50 vs. 47.00 ± 14.81 years, *P* < 0.001) and had a higher prevalence of MAFLD (47.70% vs. 30.45%, *P* < 0.001). They were also more likely to exhibit metabolic syndrome-related conditions, including diabetes (12.52% vs. 8.30%, *P* < 0.001), hypertension (48.44% vs. 33.83%, *P* < 0.001), and obesity (22.09% vs. 13.48%, *P* < 0.001). Additionally, the prevalence of nephrolithiasis was significantly higher in male participants compared to female participants (10.29% vs. 3.30%, *P* < 0.001).

**Table 1:** Demographic and clinical characteristics of participants.

Variables	Non-nephrolithiasis (n = 90,188)	Nephrolithiasis (n = 6,579)	*P*-value
Age, years	47.00 ± 14.81	49.30 ± 13.50	<0.001
Sex, n (%)			<0.001
Female	39,256 (43.53)	1,337 (20.32)	
Male	50,932 (56.47)	5,242 (79.68)	
MAFLD, n (%)			<0.001
No	62,724 (69.55)	3,441 (52.30)	
Yes	27,464 (30.45)	3,138 (47.70)	
Diabetes, n (%)			<0.001
No	82,699 (91.70)	5,755 (87.48)	
Yes	7,489 (8.30)	8,24 (12.52)	
Hypertension, n (%)			<0.001
No	59,675 (66.17)	3,392 (51.56)	
Yes	30,513 (33.83)	3,187 (48.44)	
Obesity, n (%)			<0.001
No	78,029 (86.52)	5,126 (77.91)	
Yes	12,159 (13.48)	1,453 (22.09)	
BMI, kg/m^2^	24.17 ± 3.54	25.47 ± 3.50	<0.001
Waist, cm	83.45 ± 9.68	87.52 ± 9.36	<0.001
ALT, U/L	26.65 ± 26.15	31.68 ± 29.81	<0.001
AST, U/L	22.60 ± 12.47	24.42 ± 22.20	<0.001
GGT, U/L	31.78 ± 36.13	39.67 ± 38.81	<0.001
Glu, mmol/L	5.52 ± 1.33	5.78 ± 1.69	<0.001
TC, mmol/L	4.86 ± 0.94	4.94 ± 0.94	<0.001
TG, mmol/L	1.74 ± 1.56	2.12 ± 1.84	<0.001
HDL-C, mmol/L	1.31 ± 0.33	1.22 ± 0.30	<0.001
LDL-C, mmol/L	2.76 ± 0.76	2.82 ± 0.76	<0.001
SCr, µmol/L	66.53 ± 19.83	72.64 ± 27.03	<0.001
UA, µmol/L	340.64 ± 90.33	376.06 ± 95.72	<0.001
eGFR, mL/min/1.73m^2^	94.41 ± 17.97	90.42 ± 18.24	<0.001

Values are n (%) or mean ± SD. MAFLD, metabolic associated fatty liver disease; BMI, body mass index; ALT, alanine aminotransferase; AST, aspartate aminotransferase; GGT, γ-glutamyl transpeptidase; Glu, fasting glucose; TC, total cholesterol; TG, triglycerides; HDL-C, high-density lipoprotein cholesterol; LDL-C, low-density lipoprotein cholesterol; SCr, serum creatinine; UA, uric acid; eGFR, estimated glomerular filtration rate.

### 3.2 The relationship between MAFLD and nephrolithiasis

These data were entered into a multifactorial analysis of two models to control for potential confounders. After logistic regression, the results showed a consistent relationship between MAFLD and an increased risk of nephrolithiasis in male participants. **Table 2** shows the ratio of participants stratified by sex who developed nephrolithiasis. The results indicate that MAFLD patients had an increased risk of nephrolithiasis after adjustment for possible confounders (adjusted *OR*=1.38, 95% *CI*: 1.29 to 1.47, *P <*0.001), with a significantly increased risk of nephrolithiasis in male MAFLD patients (adjusted *OR*=1.43, 95% *CI*: 1.34 to 1.54, *P*<0.001), and this association was not statistically significant in female MAFLD patients.

**Table 2:** Sex-stratified odds ratios for nephrolithiasis in patients with MAFLD.

	Odds ratio (95%*CI*) *P*-value		
	Non-adjusted	Adjust I	Adjust II
Female			
No MAFLD	reference	reference	reference
MAFLD	1.53 (1.34, 1.74) <0.001	1.27 (1.09, 1.47) 0.002	1.13 (0.9, 1.34) 0.163
Male			
No MAFLD	reference	reference	reference
MAFLD	1.67 (1.58, 1.77) <0.001	1.53 (1.43, 1.62) <0.001	1.43 (1.34, 1.54) <0.001
Total			
No MAFLD	reference	reference	reference
MAFLD	1.65 (1.56, 1.73) <0.001	1.48 (1.40, 1.57) <0.001	1.38 (1.29, 1.47) <0.001

Multivariable model 1 was adjusted for age; diabetes; hypertension; obesity.

Model 2: model 1 plus adjustment for aminotransferase, aspartate aminotransferase, γ-glutamyl transpeptidase, triglycerides, high-density lipoprotein cholesterol, low-density lipoprotein cholesterol, uric acid, and estimated glomerular filtration rate.

### 3.3 Subgroup analyzes on the association between MAFLD and nephrolithiasis

**Figure 2:**
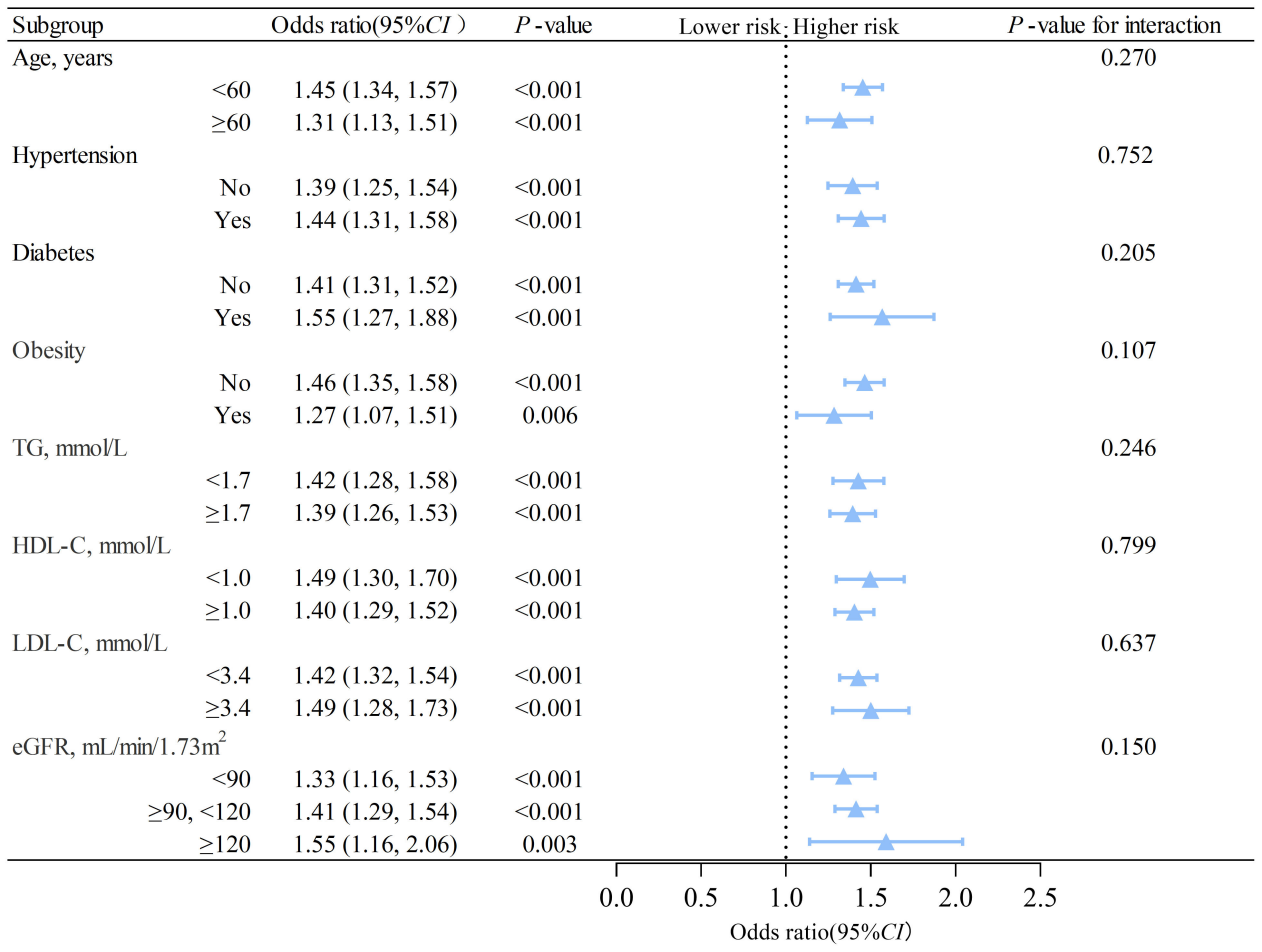
Subgroup analysis of the association between MAFLD and nephrolithiasis in males. TG, triglycerides; HDL-C, high-density lipoprotein cholesterol; LDL-C, low-density lipoprotein cholesterol; eGFR, estimated glomerular filtration rate; CI, confidence interval. The model is adjusted for variables in model 2 (as described in the statistical analysis section), except for the stratification variable.

**Figure 3:**
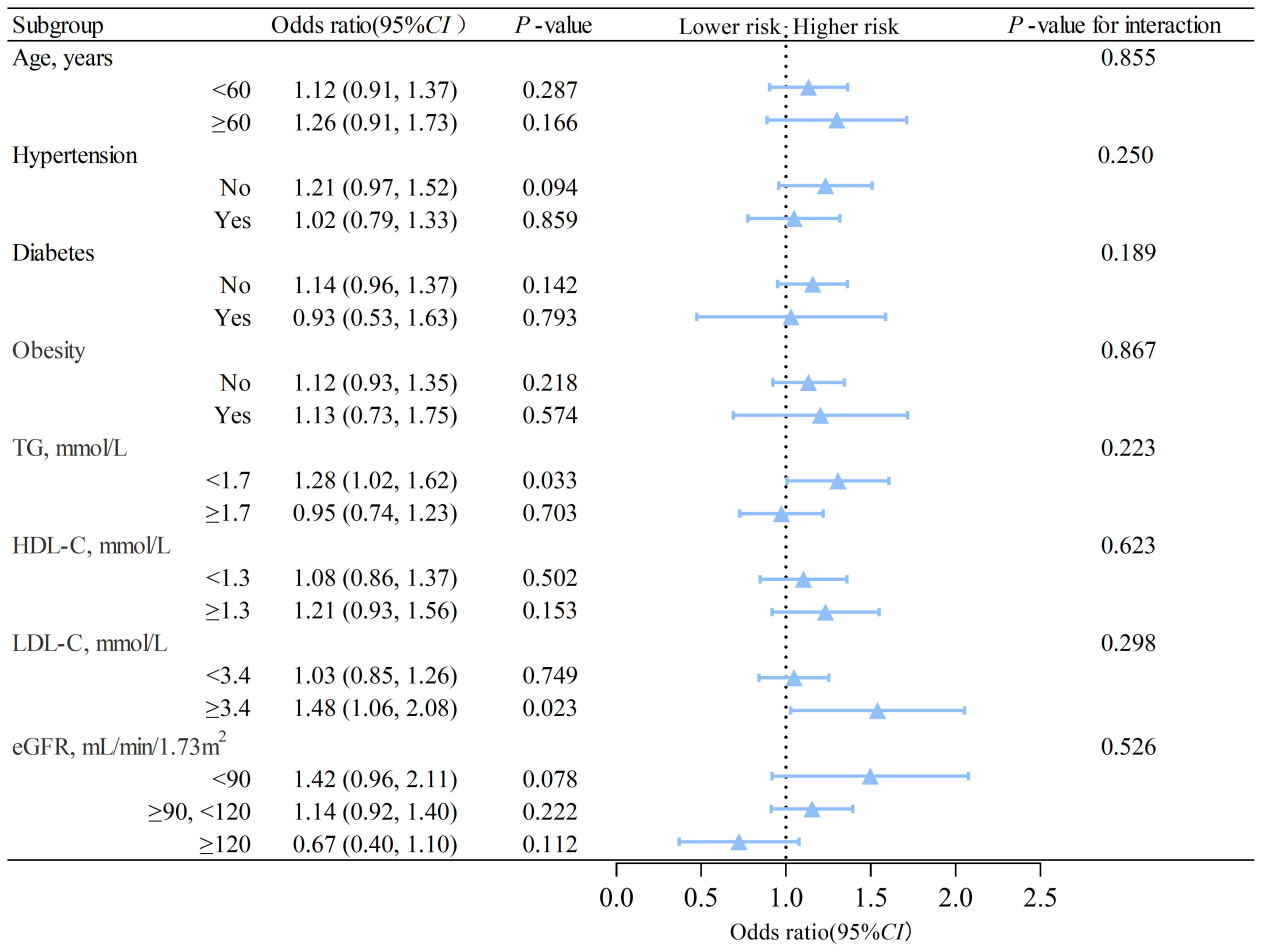
Subgroup analysis of the association between MAFLD and nephrolithiasis in females. TG, triglycerides; HDL-C, high-density lipoprotein cholesterol; LDL-C, low-density lipoprotein cholesterol; eGFR, estimated glomerular filtration rate; CI, confidence interval. The model is adjusted for variables in model 2 (as described in the statistical analysis section), except for the stratification variable.

We conducted subgroup analyzes based on sex using interaction analysis. The results showed that in males, MAFLD was linked to a higher risk of nephrolithiasis regardless of the presence of different risk factors. The *OR*s were greater than 1, and *P*s were less than 0.05 in all subgroups (**Figure 2**). In females, low triglyceride (adjusted *OR*=1.28, 95% *CI*: 1.02 to 1.62, *P*= 0.033) was associated with an elevated risk of nephrolithiasis (**Figure 3**). No significant interactions were found between risk factors and the impact of MAFLD on the risk of nephrolithiasis in our study population.

In further subgroup analysis, we found a significant increase in the severity of liver steatosis (as assessed by ultrasound images) in the MAFLD population with nephrolithiasis compared to those without nephrolithiasis, which were severe (1.06% vs. 0.46%), moderate (10.82% vs. 6.21%) and mild (35.81% vs. 23.78%) (**Supplementary Table S1**). **Table 3** shows the odds ratios for nephrolithiasis in patients with MAFLD stratified by sex and severity of liver steatosis. The results show that the risk of nephrolithiasis in male patients increased gradually with increasing severity of hepatic steatosis, with adjusted multivariate odds ratios for mild (adjusted *OR*=1.43, 95% *CI*: 1.33 to 1.53, *P*<0.001), moderate (adjusted *OR*=1.48, 95% *CI*: 1.32 to 1.67, *P*<0.001) and severe (adjusted *OR*=1.94, 95%*CI*: 1.47 to 2.58, *P*<0.001). In female patients, this relationship was not statistically significant.

**Table 3:** Sex and liver steatosis severity-stratified odds ratios for nephrolithiasis in MAFLD patients.

Severity of liver steatosis	Odds ratio (95%*CI*) *P*-value		
	Non-adjusted	Adjust I	Adjust II
Female			
No	reference	reference	reference
Mild	1.43 (1.24, 1.66) <0.001	1.22 (1.04, 1.43) 0.016	1.10 (0.92, 1.31) 0.289
Moderate	1.98 (1.53, 2.55) <0.001	1.59 (1.20, 2.11) 0.001	1.34 (0.98, 1.84) 0.063
Severe	1.74 (0.54, 5.57) 0.351	1.32 (0.41, 4.27) 0.646	1.20 (0.37, 3.92) 0.762
Male			
No	reference	reference	reference
Mild	1.63 (1.53, 1.73) <0.001	1.51 (1.41, 1.61) <0.001	1.43 (1.33, 1.53) <0.001
Moderate	1.77 (1.61, 1.94) <0.001	1.59 (1.44, 1.77) <0.001	1.48 (1.32, 1.67) <0.001
Severe	2.33 (1.79, 3.04) <0.001	2.06 (1.57, 2.70) <0.001	1.94 (1.47, 2.58) <0.001
Total			
No	reference	reference	reference
Mild	1.60 (1.51, 1.69) <0.001	1.46 (1.38, 1.55) <0.001	1.37 (1.28, 1.46) <0.001
Moderate	1.78 (1.63, 1.94) <0.001	1.58 (1.43, 1.74) <0.001	1.44 (1.29, 1.61) <0.001
Severe	2.28 (1.77, 2.95) <0.001	1.98 (1.52, 2.58) <0.001	1.84 (1.40, 2.42) <0.001

MAFLD, metabolic associated fatty liver disease.

Multivariable model 1 was adjusted for age; diabetes; hypertension; obesity.

Model 2: model 1 plus adjustment for aminotransferase, aspartate aminotransferase, γ-glutamyl transpeptidase, triglycerides, high-density lipoprotein cholesterol, low-density lipoprotein cholesterol, uric acid, and estimated glomerular filtration rate.

### 3.4 Sensitivity analysis

To evaluate the robustness of our previous findings, we performed a sensitivity analysis between populations with missing data and multiple imputation (MI). We replicated several key analyses from our prior work and compared the results to those obtained before multiple imputation. The results of **Supplementary Table S2** showed that except for waist circumference, the imputed data from other variables were highly consistent with the original data (all *P*>0.05). Although there were slight differences in the imputed waist circumference data (*P*<0.05), considering the sizeable original sample size (n=95,767) that can lead to occasional errors in individual variables, and waist circumference had the highest percentage of missing values (13,806, 14.4%), which exaggerated the imputation errors, the multiple imputation overall verified the reliability of the results by successfully retaining the general distribution characteristics of the original sample. **Supplementary Tables S3** and **S4** confirmed the consistency between the results after multiple imputation and the analysis with missing data, which validated the robustness of our research.

## 4 Discussion

The findings of this study suggest an association between MAFLD and nephrolithiasis. MAFLD increases the risk of nephrolithiasis formation. These results suggest that clinicians should be aware that MAFLD patients are at risk of nephrolithiasis and should take appropriate preventive and therapeutic measures. Furthermore, the study showed a progressive increase in the risk of nephrolithiasis in males with MAFLD as the severity of liver steatosis increased.

In 2013, Einollahi et al. (16) investigated 11,245 ultrasound reports in a cross-sectional study and found a higher detection rate of kidney stones in patients with NAFLD than in healthy controls. Subsequent studies have shown a progressive increase in the prevalence of urolithiasis with increasing severity of NAFLD. NAFLD can progress to cirrhosis and eventually to hepatocellular carcinoma, and investigations by Qin et al. (17, 18) demonstrated the impact of the progression of NAFLD on the risk of urolithiasis among patients with NAFLD. Noninvasive biomarkers of liver fibrosis, such as the APRI score and the FIB-4 score, can be used as markers to detect urolithiasis in patients with NAFLD.

However, the present study varies between regions. For example, a cohort study of Korean adults by Kim et al. (19) found that NAFLD was associated with an elevated risk of developing nephrolithiasis in men (adjusted *HR*=1.17, 95% *CI*: 1.06 to 1.30) but not in women (adjusted *HR*=0.97, 95% *CI*: 0.81 to 1.16). On the contrary, another cohort study of US adults found that NAFLD was associated with an elevated risk of nephrolithiasis in female (*OR*=1.65, 95% *CI*: 1.17 to 2.32) but not in male (*OR*=1.04, 95% *CI*: 0.77 to 1.40) (20).

The results of our study were similar to those of the Korean study, which may be because both of our study cohorts primarily consisted of individuals from East Asian backgrounds. In contrast, the US cohort was predominantly white and black racial groups. Moreover, in both our study and the Korean cohort, males exhibited a higher mean BMI than females and a greater prevalence of obesity, diabetes, and hypertension. Conversely, in the US cohort, women had a significantly higher mean BMI and greater prevalence of these metabolic conditions compared to men (19, 20).

The association of MAFLD with nephrolithiasis may arise from shared risk factors such as diabetes, hypertension, and obesity, components of MetS. MAFLD is inextricably related to MetS and its features and is considered a liver manifestation of MetS (21). Nephrolithiasis has been strongly associated with MetS, with evidence indicating a twofold increased risk of developing MetS among affected patients (22). Similarly, MetS increases the risk of nephrolithiasis, and this association grows stronger as the number of MetS components increases (23). Lifestyle factors, particularly sedentary behavior, have been shown to contribute to the progression of MAFLD and are associated with an increased risk of nephrolithiasis (24, 25).

Beyond these shared metabolic factors and lifestyle factors, sex hormones may play a pivotal role in explaining the observed disparities. Estrogen, which is predominant in females, exerts protective effects against nephrolithiasis by increasing urinary citrate levels, reducing calcium and oxalate excretion, and thereby lowering the risk of stone formation (26). Conversely, testosterone, which is more prevalent in males, has been associated with increased urinary calcium excretion, potentially elevating the risk of nephrolithiasis (27).

Differences in dietary habits and hydration patterns between sexes may also contribute to the observed disparity. In East Asian populations, high-sodium diets are particularly common among males and are associated with increased urinary calcium excretion, which raises the risk of kidney stone formation (28, 29). Conversely, females generally maintain better hydration habits, including higher water intake, which dilutes urinary solutes and lowers the risk of nephrolithiasis (30).

The physiological mechanisms between MAFLD and nephrolithiasis are unclear. IR plays a crucial role in the development and progression of hepatic steatosis. IR promotes the development of liver steatosis to steatohepatitis by promoting lipolysis of adipose tissue, the release of free fatty acids, and their deposition in the liver (31). The insulin receptor is expressed in the renal tubular epithelium. It is involved in and promotes ammonia production in the proximal tubule, preventing low urinary pH. However, the effect of IR on proximal tubular ammonia production and urinary pH is diminished, resulting in precipitation of uric acid and changes in urine composition. These factors contribute to the accumulation of calcium oxalate and uric acid stones, increasing the risk of nephrolithiasis (32). Oxidative stress (OS) is believed to be related to the pathogenesis of hepatic steatosis (33). The kidney is particularly susceptible to oxidative damage due to the rich content of long-chain polyunsaturated fatty acids in its lipid composition. It has been suggested that apoptosis and membrane-bound vesicles induced by the OS response promote crystal formation as the basis for early stone formation. The subsequent inflammatory immune response promotes the formation of Randall’s plaque and calcium oxalate stones (34).

Alterations in uric acid metabolism and inflammatory pathways provide key insights into the shared mechanisms linking MAFLD and nephrolithiasis. Hyperuricemia, a common feature of MAFLD, increases uric acid supersaturation in acidic urinary environments, facilitating crystal nucleation and serving as a nidus for stone development (35, 36). Additionally, uric acid acts as a pro-inflammatory mediator by activating the NLRP3 inflammasome, triggering the release of cytokines such as IL-1β, which exacerbate renal inflammation and tubular injury (37). MAFLD, characterized by chronic low-grade inflammation, further amplifies this pro-inflammatory state, contributing to systemic metabolic dysfunction and immune activation (38). Notably, recent studies have identified a significant association between elevated systemic immune-inflammatory index (SII) values and an increased risk of nephrolithiasis, further underscoring the pivotal role of systemic inflammation in its pathogenesis (39).

Additionally, environmental exposures have been implicated in the development of both nephrolithiasis and MAFLD. For example, phthalate metabolites, such as MiBP and MBzP, have been linked to an increased risk of nephrolithiasis, while MECPP, MEP, and MEHHP have been shown to exacerbate MAFLD through metabolic dysfunction (40, 41).

Some researchers have found that people with abnormal lipid metabolism have a reduced urine pH. On the one hand, high triglycerides in the body lead to higher concentrations of oxalate and uric acid in the urine. On the other hand, it is related to decreased ammonia synthesis and secretion due to increased lipotoxicity of fatty acids in the proximal tubules of the kidney (42, 43). Studies in animal models have shown that increased oxalate synthesis in the liver is associated with liver steatosis. Glyoxylate is a precursor of oxalic acid and is transferred to glycine catalyzed by alanine glyoxylate aminotransferase. It was found that hepatic steatosis affects the expression of this enzyme gene, leading to hypermethylation of its gene, which in turn downregulates the face of other enzyme genes associated with oxalate production. Gianmoena et al. (44) suggested that the reduced detoxification capacity of glyoxylate in patients with fatty liver leads to increased oxalate production and an increased risk of developing nephrolithiasis.

This research marks the first investigation into the connection between MAFLD and nephrolithiasis. It demonstrates that this association is more robust with increasing severity of hepatic steatosis. Subgroup analysis showed that sex was influential in the association between MAFLD and nephrolithiasis, with MAFLD being a risk factor for nephrolithiasis in the male population and a nonsignificant association with nephrolithiasis in the female population. These results help clinicians develop more precise treatment plans.

This study still has several limitations. First, our MAFLD and nephrolithiasis were based on ultrasound diagnosis. Although ultrasound diagnosis is widely used clinically as a screening method for hepatic steatosis (45), it still cannot replace the gold standard of liver biopsy, and ultrasound has a low sensitivity in detecting mild fatty liver. This type of error can lead to an underestimation of the true association between MAFLD and nephrolithiasis. Second, we did not obtain information on diet, physical activity and history of other comorbid diseases, such as hyperparathyroidism and inflammatory bowel disease, which are important risk factors for nephrolithiasis (46–48). Third, we lack analysis of the composition and location of nephrolithiasis, as well as differentiation between first-time and recurrent kidney stone cases. Fourth, the ethnic population of this study was primarily Asian. Therefore, our findings may not be directly applicable to populations from other ethnic backgrounds. Fifth, our results relied on single-center physical examination information. The retrospective data from a single center may introduce selection bias and decrease generalizability of the findings. Thus, it is difficult to fully eliminate biases inherent in the study design. Further large-scale prospective multicenter cohort studies are warranted to validate the relationship between MAFLD and risk of nephrolithiasis in broader populations. Finally, our study is a cross-sectional study and other future studies are needed to analyze the causal relationship between MAFLD and the development of nephrolithiasis.

## 5 Conclusions

In conclusion, this cross-sectional study revealed a significant association between MAFLD and nephrolithiasis in a Chinese population, particularly in male participants. The risk of nephrolithiasis increased with the severity of liver steatosis. These findings highlight the need for further research to explore the biological mechanisms linking MAFLD and nephrolithiasis and to guide effective prevention and management strategies.

## Data Availability

The raw data supporting the conclusions of this article will be made available by the authors, without undue reservation.
