# Supplementary material for: Metabolic dysfunction-associated fatty liver disease and risk of nephrolithiasis: a sizeable cross-sectional study
Source: Front Endocrinol (Lausanne). 2025 Jan 21;15:1406065. doi: 10.3389/fendo.2024.1406065 (PMC11790460; doi:10.3389/fendo.2024.1406065)
Supplement: Supplementary file 1 [file Table1.docx]

Supplementary Material

Table S1. Severity of hepatic steatosis and nephrolithiasis.

|  | Female (n = 40,593) | | Male (n = 56,174) | | All participants (n = 96,767) | |
| --- | --- | --- | --- | --- | --- | --- |
| Nephrolithiasis | No =  39,256 | Yes =  1,337 | No =  50,932 | Yes =  5,242 | No =  90,188 | Yes =  6,579 |
| Severity of hepatic steatosis | | | | | | |
| No | 32,803  (83.56%) | 1,028  (76.89%) | 29,921  (58.75%) | 2,413  (46.03%) | 62,724  (69.55%) | 3,441  (52.30%) |
| Mild | 5,318  (13.55%) | 239  (17.88%) | 16,132  (31.67%) | 2,117  (40.39%) | 21,450  (23.78%) | 2,356  (35.81%) |
| Moderate | 1,080  (2.75%) | 67  (5.01%) | 4,523  (8.88%) | 645  (12.30%) | 5,603  (6.21%) | 712  (10.82%) |
| Severe | 55  (0.14%) | 3  (0.22%) | 356  (0.70%) | 67  (1.28%) | 411  (0.46%) | 70  (1.06%) |
| *P* < 0.001. | | | | | | |

Table S2. Comparation of basic characteristics before and after multiple imputation.

| Variables | Before MI | After MI | *P*-value |
| --- | --- | --- | --- |
| Waist, cm | 83.84 ± 9.66 | 83.74 ± 9.69 | 0.031 |
| ALT, U/L | 26.99 ± 26.45 | 27.00 ± 26.45 | 0.904 |
| AST, U/L | 22.72 ± 13.37 | 22.71 ± 13.37 | 0.905 |
| GGT, U/L | 32.31 ± 36.43 | 32.36 ± 36.44 | 0.650 |
| Glu, mmol/L | 5.54 ± 1.36 | 5.54 ± 1.36 | 0.806 |
| TC, mmol/L | 4.87 ± 0.94 | 4.87 ± 0.94 | 0.401 |
| TG, mmol/L | 1.77 ± 1.60 | 1.77 ± 1.60 | 0.471 |
| HDL, mmol/L | 1.31 ± 0.33 | 1.31 ± 0.33 | 0.176 |
| LDL, mmol/L | 2.77 ± 0.75 | 2.77 ± 0.75 | 0.731 |
| Scr, μmol/L | 66.94 ± 20.53 | 66.96 ± 20.52 | 0.711 |
| UA, µmol/L | 343.17 ± 91.16 | 343.13 ± 91.14 | 0.987 |
| eGFR, mL/min/1.73m^2^ | 104.34 ± 16.37 | 104.37 ± 16.34 | 0.757 |
| MI, multiple imputation; BMI, body mass index; ALT, alanine aminotransferase; AST, aspartate aminotransferase; GGT, γ-glutamyl transpeptidase; Glu, fasting glucose; TC, total cholesterol; TG, triglycerides; HDL, high-density lipoprotein cholesterol; LDL, low-density lipoprotein cholesterol; SCr, serum creatinine; UA, uric acid; eGFR, estimated glomerular filtration rate. | | | |
| No. of participants have missing data for the variables: Waist, n=13,806; ALT, n=168; AST, n=169; GGT, n=681; Glu, n=1,657; TC, TG, n=3,212; HDL, LDL n=8,357; SCr, n=1,195; UA, n=1,200. | | | |

Table S3. Comparison of odds ratios for nephrolithiasis in patients with MAFLD by sex between original data and multiple imputation.

|  | Odds ratio (95%*CI*) *P*-value | |
| --- | --- | --- |
|  | Before MI | After MI |
| Female |  |  |
| No MAFLD | reference | reference |
| MAFLD | 1.13 (0.95, 1.34) 0.163 | 1.16 (0.98, 1.37) 0.077 |
| Male |  |  |
| No MAFLD | reference | reference |
| MAFLD | 1.43 (1.34, 1.54) <0.001 | 1.45 (1.35, 1.55) <0.001 |
| Total |  |  |
| No MAFLD | reference | reference |
| MAFLD | 1.38 (1.29, 1.47) <0.001 | 1.40 (1.31, 1.49) <0.001 |
| MAFLD, metabolic associated fatty liver disease; MI, multiple imputation.  Above OR adjusted for model 2 (as shown in statistical analysis). | | |

Table S4. Comparison of odds ratios for nephrolithiasis in patients with MAFLD stratified by sex and severity of liver steatosis between original data and multiple imputation.

|  | Odds ratio (95%CI) *P*-value | |
| --- | --- | --- |
| Severity of liver steatosis | Before MI | After MI |
| Female |  |  |
| No | reference | reference |
| Mild | 1.10 (0.92, 1.31) 0.289 | 1.13 (0.95, 1.34) 0.171 |
| Moderate | 1.34 (0.98, 1.84) 0.063 | 1.41 (1.05, 1.90) 0.023 |
| Severe | 1.20 (0.37, 3.92) 0.762 | 1.18 (0.36, 3.83) 0.784 |
| Male |  |  |
| No | reference | reference |
| Mild | 1.43 (1.33, 1.53) <0.001 | 1.44 (1.34, 1.54) <0.001 |
| Moderate | 1.48 (1.32, 1.67) <0.001 | 1.51 (1.35, 1.69) <0.001 |
| Severe | 1.94 (1.47, 2.58) <0.001 | 1.94 (1.47, 2.56) <0.001 |
| Total |  |  |
| No | reference | reference |
| Mild | 1.37 (1.28, 1.46) <0.001 | 1.38 (1.30, 1.48) <0.001 |
| Moderate | 1.44 (1.29, 1.61) <0.001 | 1.47 (1.33, 1.64) <0.001 |
| Severe | 1.84 (1.40, 2.42) <0.001 | 1.84 (1.41, 2.41) <0.001 |
| MAFLD, metabolic associated fatty liver disease; MI, multiple imputation..  Above OR adjusted for model 2 (as shown in statistical analysis). | | |

**Table Captions**

Table S1. Severity of hepatic steatosis and nephrolithiasis.

Table S2. Comparation of basic characteristics before and after multiple imputation.

Table S3. Comparison of odds ratios for nephrolithiasis in patients with MAFLD by sex between original data and multiple imputation.

Table S4. Comparison of odds ratios for nephrolithiasis in patients with MAFLD stratified by sex and severity of liver steatosis between original data and multiple imputation.
